# Occupational exposure to organic solvents and risk of bladder cancer

**DOI:** 10.1038/s41370-024-00651-4

**Published:** 2024-02-16

**Authors:** Shuai Xie, Melissa C. Friesen, Dalsu Baris, Molly Schwenn, Nathaniel Rothman, Alison Johnson, Margaret R. Karagas, Debra T. Silverman, Stella Koutros

**Affiliations:** 1 Occupational and Environmental Epidemiology Branch, Division of Cancer Epidemiology and Genetics, National Cancer Institute, National Institutes of Health, Department of Health and Human Services, Bethesda, MD USA; 2 Formerly Maine Cancer Registry, Augusta, ME USA; 3 Formerly Vermont Department of Health, Burlington, VT USA; 4 Department of Epidemiology, Geisel School of Medicine at Dartmouth, Hanover, NH USA

**Keywords:** Bladder cancer, Benzene, Toluene, Xylene, Organic solvents, Occupational exposure

## Abstract

**Background:**

Bladder cancer has been linked to several occupations that involve the use of solvents, including those used in the dry-cleaning industry.

**Objectives:**

We evaluated exposure to solvents and risk of bladder cancer in 1182 incident cases and 1408 controls from a population-based study.

**Methods:**

Exposure to solvents was quantitatively assessed using a job-exposure matrix (CANJEM). Exposure to benzene, toluene and xylene often co-occur. Therefore, we created two additional sets of metrics for combined benzene, toluene and xylene (BTX) exposure: (1) CANJEM-based BTX metrics and (2) hybrid BTX metrics, using an approach that integrates the CANJEM-based BTX metrics together with lifetime occupational histories and exposure-oriented modules that captured within-job, respondent-specific details about tasks and chemicals. Adjusted odds ratios (ORs) and 95% confidence intervals (95% CI) were estimated using logistic regression.

**Results:**

Bladder cancer risks were increased among those ever exposed to benzene (OR = 1.63, 95% CI: 1.14–2.32), toluene (OR = 1.60, 95% CI: 1.06–2.43), and xylene (OR = 1.67, 95% CI: 1.13–2.48) individually. We further observed a statistically significant exposure-response relationship for cumulative BTX exposure, with a stronger association using the hybrid BTX metrics (OR_Q1vsUnexposed_ = 1.26, 95% CI: 0.83–1.90; OR_Q2vsUnexposed_ = 1.52, 95% CI: 1.00–2.31; OR_Q3vsUnexposed_ = 1.88, 95% CI: 1.24–2.85; and OR_Q4vsUnexposed_ = 2.23, 95% CI: 1.35–3.69) (*p*-trend=0.001) than using CANJEM-based metrics (*p*-trend=0.02).

**Impact:**

There is limited evidence about the role of exposure to specific organic solvents, alone or in combination on the risk of developing bladder cancer. In this study, workers with increasing exposure to benzene, toluene, and xylene as a group (BTX) had a statistically significant exposure-response relationship with bladder cancer. Future evaluation of the carcinogenicity of BTX and other organic solvents, particularly concurrent exposure, on bladder cancer development is needed.

## Introduction

According to GLOBOCAN estimates, bladder cancer was the tenth most frequently diagnosed cancer globally in 2020 [1] and the sixth most diagnosed cancer in the United States [2]. Occupational carcinogens are estimated to be accountable for approximately 5–25% of bladder cancer among males and 8–11% among females [3]. Several occupations with likely solvent exposure have been linked to bladder cancer, including employment in the dry cleaning industry, in the rubber manufacturing industry [4, 5], as well as painters [6]. Workers in these occupations are likely to be exposed to a variety of organic solvents when they dissolve and dilute materials for their work processes [7]. Substantial exposure to benzene, toluene and xylene, in particular, can be detected in the workplaces of these ‘high-risk’ occupations [8, 9]. Perchloroethylene, a solvent used in dry-cleaning, is the only solvent with probable evidence for bladder carcinogenicity [10] but the role of other commonly used organic solvents on bladder cancer risk is still unclear and needs additional consideration.

Results of previous studies examining the association between specific organic solvents and risk of bladder cancer are inconsistent with some showing positive associations [11–13] and others showing no association [14–16]. Several studies determined exposure based on job title or assessed diverse groups of workers in certain industries, limiting the assessment of specific solvents. Recently, two studies used job-exposure matrices to capture exposure to specific solvents and to incorporate quantitative assessments of increasing exposure [11, 13]. Sciannameo et al. pooled data from two Italian case-control studies and found a positive association for trichloroethane exposure, but no exposure-response was apparent [13]. A recent large study of 14.9 million individuals from Denmark, Finland, Iceland, Norway, and Sweden found significant positive associations between heavy exposure to trichloroethylene (hazard ratio (HR) = 1.23, 95% CI: 1.12–1.40), toluene (HR = 1.20, 95% CI: 1.00–1.38) and benzene (HR = 1.16, 95% CI: 1.04–1.31) and risk of bladder cancer. This study however, did not control for cigarette smoking, the major risk factor for bladder cancer [11].

In the current study, we used data from the New England Bladder Cancer Study (NEBCS), a large population-based case-control study with detailed lifetime occupational histories as well as data on smoking and other potential confounders, to evaluate the association between occupational exposure to solvents and risk of bladder cancer.

## Materials And methods

### Study population

The NEBCS study population has been previously reported in detail [17]. Cases included individuals between the ages of 30 and 79 years who were newly diagnosed with histologically confirmed urothelial cell carcinoma of the bladder (UBC) living in Maine, Vermont, and New Hampshire between 2001–2004. Of 1878 eligible cases, 1213 (65%) were interviewed. In each state, Department of Motor Vehicle (DMV) records and Centers for Medicare and Medicaid Services (CMS) beneficiary records were used to randomly select controls aged 30–64 years and 65–79 years, respectively. Controls were frequency-matched to cases based on the state, gender, and five-year age group at diagnosis/interview. Sixty-five percent of eligible DMV controls (*n* = 594) and 65% of eligible CMS controls (*n* = 824) participated in the interview. Nonparticipation among eligible cases and controls was due to refusal, difficulty locating the participants, sickness or death, and inability to communicate in English. After excluding participants without a complete occupational history, the current analytical dataset included 1182 cases and 1408 controls (Table 1).

**Table 1:** Descriptive characteristics of bladder cancer cases and controls in the New England Bladder Cancer Study.

	Cases		Controls	
	*N* (%)		*N* (%)	
Total	1182		1408	
Age at diagnosis/interview				
<55	190	(16.07%)	254	(18.04%)
55–64	313	(26.48%)	335	(23.79%)
65–74	434	(36.72%)	541	(38.42%)
75+	245	(20.73%)	278	(19.74%)
Gender				
Female	272	(23.01%)	371	(26.35%)
Male	910	(76.99%)	1037	(73.65%)
Smoking status				
Occasional Smoker	22	(1.86%)	40	(2.84%)
Non-Smoker	174	(14.72%)	470	(33.38%)
Former Smoker	608	(51.44%)	692	(49.15%)
Current Smoker	377	(31.90%)	205	(14.56%)
Don’t Know	1	(0.08%)	1	(0.07%)
State				
Maine	575	(48.65%)	732	(51.99%)
Vermont	213	(18.02%)	251	(17.83%)
New Hampshire	394	(33.33%)	425	(30.18%)
Race				
White	1113	(94.16%)	1325	(94.11%)
Other	69	(5.84%)	83	(5.39%)
Hispanic				
Yes	23	(1.95%)	24	(1.70%)
No	1159	(98.05%)	1381	(98.08%)
Don’t Know	0	(0%)	3	(0.21%)
High-risk Occupation				
No	776	(65.65%)	1131	(80.33%)
Yes	406	(34.35%)	277	(19.67%)

All respondents gave written informed consent to participate in this study. The study protocol was approved by the National Cancer Institute, as well as the human subjects review boards of each participating institution.

### Lifetime occupational histories and solvent exposure assessment

Participants in the NEBCS reported all jobs held for at least six months after turning 16 years old, except for unpaid work and absentee ownership of businesses. For each job, participants were asked open-ended questions regarding work frequency (hours/week), the year when this job started and stopped, industry, main tasks/activities conducted, tools/equipment utilized, and chemicals/materials handled. Depending on their free-text responses, participants were administered job-and industry-specific questionnaires (module) with exposure-oriented questions on tasks and chemicals [18].

All jobs were coded by experts using the 2010 U.S. Standard Occupational Classification (SOC) system [19]. To assess solvent exposure, we used a Canadian job-exposure matrix (CANJEM) to link each job to obtain the probability of exposure and the frequency-weighted intensity (FWI) exposure estimates for 20 solvents/solvent groups (Table 2), which include broad classes of solvents, such as mononuclear aromatic hydrocarbons, and individual chemicals, such as benzene, toluene, and xylene [20]. The CANJEM version used in this study is based on SOC2010 with four calendar periods (1920–1970,1971–1980,1981–1990,1991–2005) and other parameters as default. If a job straddled time periods, we split the job into multiple records. We applied CANJEM using a hierarchical approach to assign probability and frequency-weighted intensity (FWI) metrics to each job record. Each FWI was initially assigned using a qualitative scale of low, medium, and high. These qualitative assessments were subsequently converted into numerical weighting scales of 1, 5, and 25 to generate quantitative, dimensionless scores for the exposures. We used the 6-digit, time period-specific SOC CANJEM estimate whenever possible. If a time period-specific estimate was unavailable for a 6-digit SOC code, we used the broader 1920–2005 estimate. If that was unavailable, we used time period-specific estimates at the 5-digit or 3-digit level.

**Table 2:** Exposure prevalence and odds ratios (ORs) and 95% confidence intervals (95% CI) associated with exposure to organic solvents (No lag).

	Prevalence of exposure^a^				Ever Exposed^b^
	Cases		Controls		ORs, 95% CI
	*N* (%)		*N* (%)		
Total	1182		1408		
Organic solvents	499	(42.22%)	527	(37.43%)	1.88 (0.84, 4.21)
Mononuclear aromatic hydrocarbons	368	(31.13%)	369	(26.21%)	1.84 (1.16, 2.91)
Benzene	146	(12.35%)	139	(9.87%)	1.63 (1.14, 2.32)
Toluene	101	(8.54%)	96	(6.82%)	1.60 (1.06, 2.43)
Xylene	94	(7.95%)	87	(6.18%)	1.67 (1.13, 2.48)
Styrene	32	(2.71%)	40	(2.84%)	0.94 (0.57, 1.55)
Aliphatic alcohols	135	(11.42%)	140	(9.94%)	1.45 (0.90, 2.35)
Isopropanol	60	(5.08%)	65	(4.62%)	1.15 (0.74, 1.81)
Chlorinated alkanes	45	(3.81%)	64	(4.55%)	0.88 (0.55, 1.40)
Carbon tetrachloride	17	(1.44%)	28	(1.99%)	0.77 (0.40, 1.47)
Chloroform	0		0		
Methylene chloride	6	(0.51%)	10	(0.71%)	1.35 (0.45, 4.10)
1,1,1-Trichlorethane	0		0		
Chlorinated alkenes	36	(3.05%)	51	(3.62%)	0.79 (0.48, 1.28)
Perchloroethylene	6	(0.51%)	19	(1.35%)	0.36 (0.13, 0.96)
Trichloroethylene	0		0		
Aliphatic ketones	31	(2.62%)	33	(2.34%)	1.16 (0.66, 2.05)
Aliphatic esters	29	(2.45%)	28	(1.99%)	1.18 (0.67, 2.10)
Alkanes (C5-C17)	394	(33.33%)	386	(27.41%)	1.66 (0.99, 2.79)
Alkanes (C18+)	300	(25.38%)	290	(20.60%)	1.25 (0.82, 1.92)

^a^Based on probability (50%).

^b^Odd ratio represents ever exposed versus never exposed (excludes uncertain). Adjusted for age, smoking status, state, race, ethnicity (Hispanic) and non-solvent exposed high-risk occupations for bladder cancer.

Participants were classified as: ever exposed if they held at least one job with an exposure probability of 50% or more, uncertain if they held jobs with between 0–50% exposure probability, and unexposed if they held jobs with 0% exposure probability. Cumulative exposure was calculated by multiplying the FWI (1-5-25 weighting scale) and job duration for each reported job and summing across all jobs held by the participant; jobs with a probability below the specified threshold were assigned a score of 0 in this calculation. We also conducted sensitivity analyses by classifying participants as ever exposed using alternative probability thresholds (25% or 80%).

Benzene, toluene, and xylene, all mononuclear aromatic hydrocarbons, have been extensively used as solvents in paints, stains, lacquers [21], adhesives [22], and in the petroleum [23], petrochemical [24], printing [25], and rubber [5, 26] industries. Benzene, toluene and xylene exposures often occur concurrently and the individual agents are highly correlated [11, 27]. Thus, in addition to the above CANJEM agents, we created two metrics to evaluate exposure to the benzene, toluene and xylene as a group. First, we developed a set of exposure metrics (probability and FWI) that combined benzene, toluene, and xylene (BTX) using the CANJEM metrics (CANJEM-based BTX metric). The probability of exposure to BTX was the highest probability level observed among the three agents. We assigned the highest FWI of the agent(s) with a probability ≥50% as the FWI of BTX. Second, we developed another set of BTX exposure metrics by modifying the CANJEM probability and intensity estimates using expert assessment to account for within-job, participant-specific heterogeneity in exposure (as described in Friesen et al. [28]). Briefly, two experts, blinded to case-control status, reviewed CANJEM-assigned exposure probability and FWI to each job/time-period specific record in conjunction with additional participant-specific information from free-text responses and modules for solvent-related tasks, tools, and chemicals. For example, we upgraded the exposure probability if there was substantial evidence of BTX exposure in a job that CANJEM suggested had a less than 50% probability. We downgraded the exposure probability if the participant specifically stated they did not use chemicals. During the expert review process, we used participant-level frequency information when available to calculate the FWI. We used the derived CANJEM-based BTX metrics, and hybrid BTX metrics to obtain participant-specific ever exposure and cumulative exposure to BTX as described above.

### Statistical analysis

We used logistic regression to calculate odds ratios (ORs) and 95% confidence intervals (CIs) for the association between ever exposure to a given solvent (as well as the combined BTX variables) and bladder cancer. Lagged estimates of exposure were used for the evaluation of exposure-response relationships to eliminate the effect of recent exposures unlikely related to cancer incidence. The Akaike Information Criteria (AIC) was used to determine which lagged estimates provided the best fit (20-year lag). Participants were categorized as: unexposed, with uncertain exposure, or exposed (probability ≥50%), with the exposed further cut into quartiles based on the distribution of unlagged exposure among controls. For a direct comparison of the CANJEM-based and hybrid BTX metrics, we also present results based on a common set of cut-points (based on the distribution of unlagged exposure among controls for the CANJEM-based BTX metric). The linear trend was examined by applying the Wald test to the midpoint of each category based on the controls without including the uncertain group. We further analyzed CANJEM-based and hybrid BTX metrics using 5-knot regression splines to explore potential non-linearity effects. Models were adjusted for age at diagnosis/interview, smoking status (never, former, current), state, race, ethnicity (Hispanic), and non-solvent exposed high-risk occupations for bladder cancer [18, 29].The correlation between cumulative exposure to benzene, toluene, and xylene was evaluated using the Spearman correlation statistic; correlation analyses were conducted among all controls and among controls who had ever been exposed to organic solvents.

## Results

Table 1 shows the characteristics of 1182 cases and 1408 controls in this study. Seventy seven percent of the study participants were male, and about half were from Maine. Study participants were predominantly non-Hispanic white, and most were older than 65 years old. The distribution of the demographic characteristics was similar between cases and controls except for smoking. As expected since smoking is the main risk factor for bladder cancer, a higher proportion of cases were current smokers compared to controls (31.9% and 14.6%, respectively). and a lower proportion of cases were nonsmokers than controls (14.7% and 33.4%, respectively). The proportion of participants working in non-solvent exposed high-risk occupations for bladder cancer was also higher among the cases (34.3%) compared to controls (19.7%).

We estimated the risk of bladder cancer for participants who were ever exposed to different solvents (Table 2). None of the participants were exposed to chloroform, 1,1,1-trichloroethane, or trichloroethylene. We observed statistically significant increased risks for exposure to mononuclear aromatic hydrocarbons overall (OR = 1.84, 95% CI: 1.16–2.91) and for the individual agents in this category, benzene (OR = 1.63, 95% CI: 1.14–2.32), toluene (OR = 1.60, 95% CI: 1.06–2.43) and xylene (OR = 1.67, 95% CI: 1.13–2.48), but not for styrene (OR = 0.94, 95% CI: 0.57–1.55). No excess risks of bladder cancer were observed for participants ever exposed to any other organic solvents. Participants ever exposed to perchloroethylene had an OR of 0.36 (95% CI: 0.13–0.96), but this point estimate was based on only 6 cases and 19 controls.

Table 3 shows the ORs and 95% CIs for unlagged and 20-year lagged cumulative exposure to benzene, toluene, and xylene based on the exposure assessment from CANJEM. Statistically significant increased risks were observed in some, but not all, quartiles for benzene (OR_Q3vsUnexposed_ lagged 20-years = 2.07, 95% CI: 1.22–3.53), toluene (OR_Q2vsUnexposed_ lagged 20-years = 2.91, 95% CI: 1.57–5.39) and xylene (OR_Q2vsUnexposed_ lagged 20-years = 2.37, 95% CI: 1.30–4.31); however, no significant trends in risk with increasing exposure were observed. No association between cumulative exposure to other solvents and risk of bladder cancer were apparent.

**Table 3:** Odds ratios (ORs) and 95% confidence intervals (CIs) for cumulative exposure to mononuclear aromatic hydrocarbons, benzene, toluene and xylene and risk of bladder cancer.

	No lag			Lagged 20-Years		
	Cases	Controls	ORs^a^ (95%CI)	Cases	Controls	ORs^a^ (95%CI)
Mononuclear aromatic hydrocarbons						
None	36	74	Ref	69	124	Ref
Q1	89	93	1.83 (1.08, 3.12)	92	94	1.64 (1.04, 2.58)
Q2	89	92	1.78 (1.05, 3.03)	89	87	1.80 (1.14, 2.85)
Q3	107	92	2.17 (1.28, 3.67)	97	91	1.86 (1.18, 2.93)
Q4	83	92	1.60 (0.94, 2.72)	60	65	1.57 (0.95, 2.60)
*p-trend*			*0.88*			*0.48*
Benzene						
None	115	189	Ref	152	239	Ref
Q1	39	35	1.72 (0.99, 2.99)	35	30	1.71 (0.97, 3.02)
Q2	33	37	1.44 (0.83, 2.51)	24	32	1.28 (0.70, 2.33)
Q3	43	33	1.99 (1.15, 3.43)	44	33	2.07 (1.22, 3.53)
Q4	31	34	1.40 (0.79, 2.48)	26	28	1.38 (0.75, 2.54)
*p-trend*			*0.25*			*0.14*
Toluene						
None	86	139	Ref	133	220	Ref
Q1	16	24	0.98 (0.47, 2.05)	18	28	0.97 (0.49, 1.90)
Q2	32	24	2.35 (1.24, 4.45)	36	22	2.91 (1.57, 5.39)
Q3	27	24	1.51 (0.79, 2.90)	23	24	1.47 (0.77, 2.81)
Q4	26	24	1.62 (0.84, 3.13)	16	17	1.45 (0.67, 3.12)
*p-trend*			*0.15*			*0.25*
Xylene						
None	128	220	Ref	169	280	Ref
Q1	17	22	1.21 (0.59, 2.50)	19	21	1.36 (0.68, 2.74)
Q2	32	22	2.40 (1.28, 4.50)	35	24	2.37 (1.30, 4.31)
Q3	19	22	1.43 (0.72, 2.86)	15	22	1.07 (0.52, 2.20)
Q4	26	21	1.65 (0.86, 3.18)	17	14	1.63 (0.75, 3.58)
*p-trend*			*0.11*			*0.18*

^a^Adjusted for age, smoking status, state, race, ethnicity (Hispanic) and non-solvent exposed high-risk occupations for bladder cancer.

In our study, exposure to benzene, toluene, and xylene were strongly correlated. Among all controls who had ever been exposed to organic solvents, the Spearman correlation between benzene and toluene was 0.62, between benzene and xylene was 0.65, and between toluene and xylene was 0.94 (Supplemental Table 1). Because of these correlations and their observed co-occurrence in the workplace, we assessed ever exposure and cumulative exposure to BTX as a group using the CANJEM-based BTX metrics and hybrid BTX metrics. Approximately 15% of the participants grouped as uncertain exposure in the CANJEM-based BTX metrics were reclassified as either non-exposed or exposed at different levels in the hybrid BTX metrics (Table 4). The CANJEM-based and hybrid BTX metrics identified 13% and 24% of the participants, respectively, as having a high probability of BTX exposure. CANJEM-based and hybrid BTX metrics had moderate agreement at the subject-level (probability: kappa = 0.62; cumulative metrics: Spearman correlation=0.61). The prevalence of BTX exposed jobs, by decade, over the occupational life history of participants is presented in Supplemental Table 2.

**Table 4:** Odds ratios (ORs) and 95% confidence intervals (CIs) for ever exposure and cumulative exposure to combined benzene, toluene and xylene (BTX) exposure from job-exposure matrix (CANJEM-based BTX metrics) and with additional expert review (hybrid BTX metrics) and risk of bladder cancer.

	CANJEM-based BTX metrics							Hybrid BTX metrics					
	No lag				Lagged 20-Years			No lag			Lagged 20-Years		
	Ca	Co	ORs^a^ (95%CI)		Ca	Co	ORs^a^ (95%CI)	Ca	Co	ORs^a^ (95%CI)	Ca	Co	ORs^a^ (95%CI)
**Ever exposed**													
None	68	121	Ref	112		188	Ref	87	156	Ref	126	224	Ref
Uncertain	939	1118	1.44 (1.03, 2.01)	915		1071	1.44 (1.09, 1.90)	802	931	1.47 (1.09, 1.99)	785	889	1.53 (1.18, 1.99)
Exposed	175	169	1.83 (1.23, 2.71)	155		149	1.78 (1.25, 2.54)	293	321	1.61 (1.15, 2.26)	271	295	1.63 (1.20, 2.21)
**Cumulative exposure**													
None	68	121	Ref	112		188	Ref	87	156	Ref	126	224	Ref
Q1	42	43	1.67 (0.96, 2.93)	40		41	1.54 (0.90, 2.63)	70	103	1.18 (0.76, 1.83)	71	97	1.26 (0.83, 1.90)
Q2	37	42	1.68 (0.96, 2.96)	32		38	1.66 (0.95, 2.90)	70	85	1.48 (0.95, 2.31)	68	82	1.52 (1.00, 2.31)
Q3	47	42	1.99 (1.15, 3.43)	44		38	1.94 (1.14, 3.30)	81	72	1.94 (1.25, 3.02)	80	76	1.88 (1.24, 2.85)
Q4	49	42	1.95 (1.14, 3.36)	39		32	2.03 (1.16, 3.57)	72	61	2.08 (1.31, 3.31)	52	40	2.23 (1.35, 3.69)
*p-trend*			*0.06*				*0.02*			*0.0014*			*0.0010*

*Ca* cases, *Co* controls.

*Adjusted for age, smoking status, state, race, ethnicity (Hispanic) and non-solvent exposed high-risk occupations for bladder cancer.

**Fig. 1:**
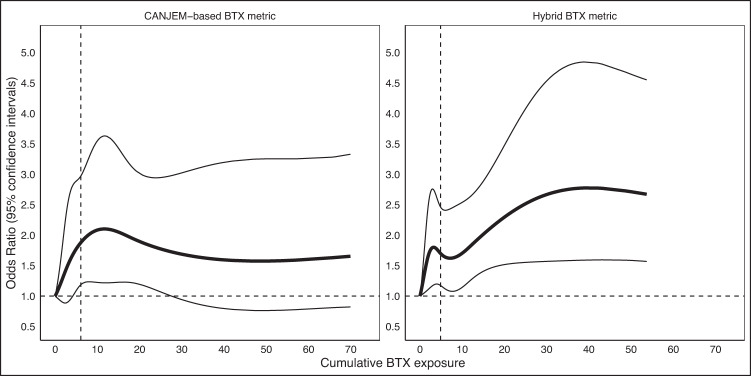
The association between cumulative BTX exposure and the risk of bladder cancer using 5-knot regression splines at 50% probability for 20-year lag. Adjusted for age, smoking status, state, race, ethnicity (Hispanic) and non-solvent exposed high-risk occupations for bladder cancer. (Vertical dash lines represent the median cumulative BTX exposure among exposed controls; solid lines represent odds ratio and 95% confidence intervals).

CANJEM-based BTX cumulative exposure was significantly associated with bladder cancer at each level (OR_Q1vsUnexposed_ lagged 20-years = 1.54, 95% CI: 0.90–2.63; OR_Q2vsUnexposed_ lagged 20-years = 1.66, 95% CI: 0.95–2.90; OR_Q3vsUnexposed_ lagged 20-years = 1.94, 95% CI: 1.14–3.30; and OR_Q4vsUnexposed_ lagged 20-years = 2.03, 95% CI: 1.16–3.57, respectively, *p*-trend = 0.02). We also observed a significant association between bladder cancer and hybrid BTX cumulative exposure (OR_Q1vsUnexposed_ lagged 20-years = 1.26, 95% CI: 0.83–1.90; OR_Q2vsUnexposed_ lagged 20-years = 1.52, 95% CI: 1.00–2.31; OR_Q3vsUnexposed_ lagged 20-years = 1.88, 95% CI: 1.24–2.85; and OR_Q4vsUnexposed_ lagged 20-years = 2.23, 95% CI: 1.35–3.69, respectively), with a more significant linear trend (*p*-trend = 0.001) compared to the CANJEM-based BTX metric, Results using the distribution of the controls for the hybrid BTX metric also showed a significant exposure-response (*p*-trend = 0.0002, Supplemental Table 3). For both CANJEM-based BTX and hybrid BTX, the 5-knot regression splines results supported the results of the quartile-based analysis (Fig. 1) and demonstrate the improvement of the exposure-response for the hybrid BTX metric, particularly at higher levels of cumulative exposure. Results considering exposure using alternate thresholds for probability (25% or 80%) are presented in Supplemental Table 4.

## Discussion

We found a statistically significant association between the risk of bladder cancer and occupational exposure to benzene, toluene, and xylene. Because exposure to these three solvents occurred concurrently, we assessed the combined effects of BTX. We found a significant exposure-response relationship between BTX and bladder cancer risk after adjustment for smoking and considering the latency for bladder cancer.

Bladder cancer risks were significantly increased among those exposed to benzene, toluene, and xylene. Our findings are consistent with the results of a recent population-based case-control study in Nordic countries where exposure to benzene and toluene at a high level significantly increased the risk of bladder cancer with a HR of 1.16 (95% CI: 1.04–1.31) and 1.20 (95% CI: 1.00–1.38), respectively [11]. Xylene was not evaluated in this study; however, the investigators noted that BTX exposure likely occurred together. In a case-cohort study of Norwegian offshore petroleum workers, workers exposed to benzene for more than 18.5 years and workers with the highest cumulative benzene exposure had 1.91 times (95% CI: 1.15–3.16; *p*-trend = 0.041) and 1.60 times (95% CI: 0.97–2.63; *p*-trend = 0.065) higher risk of bladder cancer than unexposed workers, respectively [30]. The International Agency for Research on Cancer (IARC) classifies benzene as a Group 1 human carcinogen based on findings for leukemia but found insufficient evidence for an association between exposure and bladder cancer [31]. This evaluation was based on inconsistent evidence largely from industry-based studies of bladder cancer mortality with few observed deaths [31]. Similarly, results from studies of toluene and bladder cancer are equivocal [13, 15]. Xylene and toluene are classified as Group 3 (not classifiable as to its carcinogenicity to humans) by IARC [32]. Our findings taken together with those of Hadkhale et al. and Shala et al., provide important new data using quantitative estimates of exposure linking benzene, toluene and xylene to risk of bladder cancer.

In our population-based study, it was evident that exposure to BTX co-occurred, consistent with reports from several studies [11, 15, 27]. In assessments of these solvent, the IARC Working Group observed that the majority of exposed workers likely had co-exposure to benzene, toluene and xylene, and that it was difficult to precisely separate their individual effects [31, 32]. Benzene, together with toluene and xylene, can be extensively used as solvents in paints, inks, dyes, thinners, adhesives, and coatings in the printing, painting, and rubber industries. Additionally, BTX produced from petroleum is used as an additive in gasoline, which poses a significant occupational risk to service station staff, drivers, and motor vehicle mechanics and repairmen [27]. In our study population, BTX exposure was frequent among shoe machine operators and tenders, automobile mechanics, and miscellaneous textile machine operators and tenders (Supplemental Table 5). Thus, we developed metrics to assess joint exposure to reflect real-life exposure scenarios. In addition, we developed hybrid BTX metrics that drew on direct questionnaire responses that indicated use of solvent. Although the hybrid BTX and CANJEM-based BTX metrics were moderately correlated (Spearman correlation_Lagged 20-Years_ = 0.58, Supplemental Table 6), we observed a statistically significant exposure-response relationship between cumulative BTX exposure and bladder cancer, with the hybrid BTX metrics (p-trend_Lagged 20-Years_ = 0.0010) showing a stronger exposure-response relationship than CANJEM-based BTX metrics (*p*-trend_Lagged 20-Years_ = 0.02), highlighting the importance of using the additional respondent-specific information from free-text responses and modules for solvent-related tasks, tools, and chemicals. We are not aware of other studies of bladder cancer that have evaluated this relationship; however, BTX as a group has been studied recently in relation to risk of cancers of the prostate and lung [27, 33] because of the increased recognition of coexisting exposure.

Previous studies conducted primarily among gas station employees have found that BTX is genotoxic, increasing the likelihood of chromosomal abnormalities [34] and lowering serum total-superoxide dismutase (T-SOD) and glutathione (GSH), which could result in DNA strand breaks [35]. Reduced levels of poly(ADP-ribose) polymerase 1 (PARP1) and DNA methyltransferases (DNMTs) among decorators and painters suggest that occupational BTX exposure may also affect DNA repair or cause genomic instability [36]. Additionally, occupational exposure to BTX may cause oxidative stress, as indicated by the lower serum levels of malondialdehyde (MDA) and 8-hydroxydeoxyguanosine (8-OHdG) in refueling workers [35], and oxidative DNA damage as a result of the intermediate metabolites creating reactive oxygen species (ROS), which could oxidize the DNA [37]. Workers exposed to BTX also had a greater risk of immune system impairment with a low CD4/CD8 ratio [38] and elevated pro-inflammatory cytokines [39].

Benzene is the only component of BTX that is an established human carcinogen [31, 32]. It has also been shown that benzene and many of its metabolic products are excreted in the urine and thus bioavailable to the bladder [40–42]. Further, many of the above biological effects ascribed to BTX exposure in studies discussed above are found in the large body of literature on experimental investigations of benzene and cross-sectional molecular epidemiological studies of populations with substantial occupational exposure to benzene [31, 43], either in the absence of toluene and xylene or after adjustment [41, 44]. These include reducing the CD4 + /CD8 +  ratio, altering levels of important cytokines, generating ROS, and causing multiple types of chromosomal aberrations [43]. The latter findings in particular are consistent with, although not specific to, genomic alterations detected in bladder tumors [45].

IARC has designated perchloroethylene and occupational exposure in dry cleaning to be probably carcinogenic to bladder cancer [46]. Perchloroethylene, the main solvent used in dry cleaning, may be responsible for the elevated risk of bladder cancer among dry cleaners. In a recent population-based case-control study, Hadkhale et al. observed a suggestive association between exposure to perchloroethylene and an increased risk of bladder cancer [11]. In our study, based on CANJEM using a 50% probability threshold, we identified only 6 perchloroethylene-exposed cases and no trichloroethylene exposed cases. Therefore, we were unable to assess bladder cancer risk for exposure to these chlorinated solvents. The most notable strength of this study is the approach to exposure assessment, which evaluated BTX as a group and modified quantitative, dimensionless exposure measures on a participant level based on detailed job- and industry-specific exposure-oriented modules, minimizing exposure misclassification. By taking subject-specific responses into consideration, this hybrid approach identified within-job exposure heterogeneity that was not captured by using only a JEM [47]. These modifications resulted in a more significant exposure-response relationship between BTX and bladder cancer.

This study is one of the most comprehensive population-based case-control studies to evaluate the association between bladder cancer risk and exposure to organic solvents with adjustment for smoking and control for known bladder cancer risk factors. Our models were conducted with the adjustment for prior employment in high-risk occupations and for exposures associated with bladder cancer in this study population (including occupational diesel engine exhaust and metalworking fluid exposures, which had no impact on the presented risks). Among previous studies that found a statistically increased risk of bladder cancer and benzene exposure, only two studies were adjusted for smoking [30, 48]. Our study also includes occupational exposure among females, addressing a critical gap left by some previous studies that only enrolled male participants (Supplemental Table 7, 8). Additionally, the latency for the development of bladder cancer is typically quite long, spanning several decades after exposure and we were able to take that into account in our analyses.

Several limitations of our study should also be recognized. Our study is a population-based case-control study, covering a wide variety of industries. It is important to recognize that there may be some variability in industrial processes and chemicals among countries when applying CANJEM to other populations. Because of their proximity and the similarly modernized occupational processes in the US and Canada, we expect comparable exposure profiles within occupations and industries, making the application of CANJEM valuable in this setting [49]. The hybrid BTX approach additionally integrates subject-specific patterns, further accounting for possible geographic differences in work tasks and exposures that may differ between Canada and the US. Our analysis was limited in disentangling the effects of exposure of benzene, toluene, and xylene individually due to their high correlation of occurrence. Elevated risks were also observed with the mononuclear aromatic hydrocarbons exposure metric, of which benzene, toluene and xylene is a subset; however, no trends were observed across exposure categories, which suggests that the risks are more specific to BTX than to all agents within the mononuclear aromatic hydrocarbons class. Ultimately, we found that evaluating BTX as a group was an approach that yielded a strong association, highlighting the importance of assessing the exposure to these solvents simultaneously, as they occur in the workplace. Although few prior studies have implicated BTX or benzene, toluene, and xylene individually as risk factors for bladder cancer, the observed increased risks here are driven by participants with reported occupations that have well-established higher risks for bladder cancer (i.e., mechanics, painters, machine operators, Supplemental Table 5) and are further supported by participant-specific indications of exposure to BTX from the detailed modules. In addition, we used the 1–5–25 weighting scales to represent a quantitative, dimensionless gradient in exposure levels for all organic solvents. While this approach offers a suitable approximation of the relative importance of low, medium, and high exposure in the majority of exposure scenarios [20], exposure measurement information is preferable. Because the NEBCS was not designed to specifically study exposure to organic solvents, as these exposures have not been previously recognized as potential bladder carcinogens, we were precluded from performing a more detailed assessment. Finally, although we cannot rule out the potential for recall bias in this case-control study, it is unlikely that participants would be aware of this association, further minimizing the potential impact for differential reporting of solvent exposures among cases compared to controls. Still, these results require confirmation in additional studies.

## Conclusion

In summary, the relationship between bladder cancer and cumulative exposure to BTX (benzene, toluene, xylene, as a group) was observed for the first time. The identification of an increased risk for these solvents highlights a little recognized occupational exposure that may play a role in the etiology of bladder cancer.

### Supplementary Information

Supplemental Tables

## Data Availability

The data from the current study are available from the corresponding author on reasonable request.
