# Supplementary material for: Occupational exposure to organic solvents and risk of bladder cancer
Source: J Expo Sci Environ Epidemiol. 2024 Feb 16;34(3):546–53. doi: 10.1038/s41370-024-00651-4 (PMC11222140; doi:10.1038/s41370-024-00651-4)
Supplement: Supplementary file 1 — Supplemental Tables [file 41370_2024_651_MOESM1_ESM.docx]

**Occupational exposure to organic solvents and risk of bladder cancer**

Shuai Xie^1^, Melissa C. Friesen^1^, Dalsu Baris^1^, Molly Schwenn^2^, Nathaniel Rothman^1^, Alison Johnson^3^, Margaret R. Karagas^4^, Debra T. Silverman^1^, Stella Koutros^1^

^1^ Occupational and Environmental Epidemiology Branch, Division of Cancer Epidemiology and Genetics, National Cancer Institute, National Institutes of Health, Department of Health and Human Services, Bethesda, Maryland, USA

^2^ Formerly Maine Cancer Registry, Augusta, Maine, USA

^3^ Formerly Vermont Department of Health, Burlington, Vermont, USA

^4^ Department of Epidemiology, Geisel School of Medicine at Dartmouth, Hanover, NH, USA

Supplemental Table 1. The correlation between benzene, toluene, and xylene exposure at 50% probability

|  |  | Benzene | Toluene | Xylene |
| --- | --- | --- | --- | --- |
| Restrict to controls, all agent pairings (N=1408). | Benzene | 1 |  |  |
|  | Toluene | 0.56 | 1 |  |
|  | Xylene | 0.58 | 0.95 | 1 |
|  |  |  |  |  |
| Restrict to controls with “organic solvent probability >0”, all agent pairings (N=527). |  | Benzene | Toluene | Xylene |
|  | Benzene | 1 |  |  |
|  | Toluene | 0.62 | 1 |  |
|  | Xylene | 0.65 | 0.94 | 1 |
|  |  |  |  |  |
| Restrict to controls for following benzene, toluene, and xylene pairings.  In each pairing restrict to controls and for participants that have cumulative exposure to either one >0 (e.g., solvent 1 OR solvent 2 >0). |  | Benzene | Toluene | Xylene |
|  | Benzene | 1 |  |  |
|  |  |  |  |  |
|  | Toluene | 0.28 | 1 |  |
|  |  | N = 169 |  |  |
|  | Xylene | 0.33 | 0.74 | 1 |
|  |  | N = 161 | N = 96 |  |

Supplemental Table 2. The prevalence of exposed jobs by decade of first exposure to combined benzene, toluene and xylene (BTX) at ≥50% probability

|  | CANJEM-BTX metrics | |  | Hybrid BTX metrics | |
| --- | --- | --- | --- | --- | --- |
|  | Number of jobs  among Controls | Number of jobs  among Cases |  | Number of jobs  among Controls | Number of jobs  among Cases |
| <1950 | 30 | 35 |  | 52 | 49 |
| 1950s | 40 | 52 |  | 95 | 98 |
| 1960s | 34 | 38 |  | 70 | 69 |
| 1970s | 30 | 26 |  | 59 | 48 |
| 1980s | 22 | 10 |  | 36 | 15 |
| 1990s | 12 | 14 |  | 7 | 13 |
| 2000s | 1 | 0 |  | 2 | 1 |

| Supplemental Table 3. Odds ratios (ORs) and 95% confidence intervals (CIs) for cumulative exposure to combined benzene, toluene and xylene (BTX) exposure from hybrid BTX metrics and risk of bladder cancer (probability≥50%) based on the distribution of the controls for the hybrid BTX metric | | | | | | | | | | |
| --- | --- | --- | --- | --- | --- | --- | --- | --- | --- | --- |
|  |  | No lag | | | |  | 20-Year | | | |
|  |  | Ca | Co | ORs* | (95% CI) |  | Ca | Co | ORs* | (95% CI) |
| None | | 87 | 156 | Ref |  |  | 126 | 224 | Ref |  |
| Q1 | | 51 | 81 | 1.12 | (0.69,1.80) |  | 52 | 80 | 1.12 | (0.71,1.75) |
| Q2 | | 69 | 80 | 1.50 | (0.96,2.35) |  | 70 | 75 | 1.65 | (1.08,2.52) |
| Q3 | | 73 | 80 | 1.53 | (0.98,2.39) |  | 74 | 81 | 1.58 | (1.04,2.40) |
| Q4 | | 100 | 80 | 2.27 | (1.48,3.47) |  | 75 | 59 | 2.34 | (1.51,3.64) |
|  | *p-trend* |  |  | *0.0002* |  |  |  |  | *0.0002* |  |

Ca: cases, Co: controls

*Adjusted for age, smoking status, state, race, ethnicity (Hispanic) and non-solvent exposed high-risk occupations for bladder cancer.

Supplemental Table 4. Odds ratios (ORs) and 95% confidence intervals (CIs) for ever exposure to combined benzene, toluene and xylene (BTX) exposure from job-exposure matrix and risk of bladder cancer

|  |  | No lag | | | |  | Lagged 20-Years | | | |
| --- | --- | --- | --- | --- | --- | --- | --- | --- | --- | --- |
| CANJEM-BTX | | Ca | Co | ORs*(95%CI) | |  | Ca | Co | ORs*(95%CI) | |
| **Probability≥25%** | | |  |  |  |  |  |  |  |  |
|  | None | 68 | 121 | Ref | |  | 112 | 188 | Ref | |
|  | Uncertain | 782 | 915 | 1.47 (1.05, 2.06) | |  | 774 | 887 | 1.48 (1.12, 1.95) | |
|  | Exposed | 332 | 372 | 1.53 (1.06, 2.19) | |  | 296 | 333 | 1.49 (1.09, 2.03) | |
|  |  |  |  |  | |  |  |  |  |  |
| **Probability≥80%** | | |  |  | |  |  |  |  |  |
|  | None | 68 | 121 | Ref | |  | 112 | 188 | Ref | |
|  | Uncertain | 1034 | 1209 | 1.47 (1.05, 2.04) | |  | 994 | 1144 | 1.47 (1.11, 1.93) | |
|  | Exposed | 80 | 78 | 1.75 (1.10, 2.78) | |  | 76 | 76 | 1.67 (1.09, 2.57) | |

Ca: cases, Co: controls

*Adjusted for age, smoking status, state, race, ethnicity (Hispanic) and non-solvent exposed high-risk occupations for bladder cancer.

Supplemental Table 5. Top 10 jobs with most total cumulative exposure for benzene, toluene, xylene, CANJEM-based BTX metrics, and hybrid BTX metrics at 50% probability

| SOC80  Code | | SOC title | Total  Cumulative  Exposure | Total  # Jobs |
| --- | --- | --- | --- | --- |
| **Benzene** | |  |  |  |
|  | 7656 | Shoe machine operators and tenders | 440 | 32 |
|  | 7659 | Miscellaneous textile machine operators and tenders | 422 | 42 |
|  | 6111 | Automobile mechanics | 369 | 178 |
|  | 7720 | Assemblers | 273 | 159 |
|  | 7661 | Cementing and gluing machine operators and tenders | 263 | 13 |
|  | 7678 | Slicing and cutting machine operators and tenders | 234 | 33 |
|  | 7679 | Miscellaneous machine operators and tenders, NEC | 233 | 94 |
|  | 5123 | Firefighting occupations | 228 | 35 |
|  | 6854 | Shoemakers and leather workers and repairers | 227 | 16 |
|  | 7752 | Hand sewing occupations | 218 | 24 |
|  |  |  |  |  |
| **Toluene** | |  |  |  |
|  | 7656 | Shoe machine operators and tenders | 429 | 32 |
|  | 7659 | Miscellaneous textile machine operators and tenders | 386 | 42 |
|  | 6462 | Carpet and soft tile installers | 329 | 5 |
|  | 6115 | Automotive body and related repairers | 300 | 41 |
|  | 7661 | Cementing and gluing machine operators and tenders | 260 | 13 |
|  | 8213 | Truck drivers, heavy | 234 | 129 |
|  | 7679 | Miscellaneous machine operators and tenders, NEC | 217 | 94 |
|  | 7759 | Miscellaneous hand working occupations | 216 | 43 |
|  | 6854 | Shoemakers and leather workers and repairers | 204 | 16 |
|  | 7678 | Slicing and cutting machine operators and tenders | 201 | 33 |
|  |  |  |  |  |
| **Xylene** | |  |  |  |
|  | 6115 | Automotive body and related repairers | 278 | 41 |
|  | 7656 | Shoe machine operators and tenders | 131 | 32 |
|  | 7659 | Miscellaneous textile machine operators and tenders | 127 | 42 |
|  | 7720 | Assemblers | 87 | 159 |
|  | 7679 | Miscellaneous machine operators and tenders, NEC | 85 | 94 |
|  | 7661 | Cementing and gluing machine operators and tenders | 83 | 13 |
|  | 7752 | Hand sewing occupations | 73 | 24 |
|  | 7669 | Coating, painting, and spraying machine operators and tenders | 73 | 13 |
|  | 7759 | Miscellaneous hand working occupations | 71 | 43 |
|  | 7678 | Slicing and cutting machine operators and tenders | 71 | 33 |
|  |  |  |  |  |
| **CANJEM-based BTX metrics** | | |  |  |
|  | 7656 | Shoe machine operators and tenders | 523 | 32 |
|  | 7659 | Miscellaneous textile machine operators and tenders | 487 | 42 |
|  | 6111 | Automobile mechanics | 406 | 178 |
|  | 6462 | Carpet and soft tile installers | 329 | 5 |
|  | 6115 | Automotive body and related repairers | 319 | 41 |
|  | 7661 | Cementing and gluing machine operators and tenders | 317 | 13 |
|  | 7679 | Miscellaneous machine operators and tenders, NEC | 290 | 94 |
|  | 7720 | Assemblers | 283 | 159 |
|  | 7678 | Slicing and cutting machine operators and tenders | 266 | 33 |
|  | 6854 | Shoemakers and leather workers and repairers | 264 | 16 |
|  |  |  |  |  |
| **Hybrid BTX metrics** | | |  |  |
|  | 6111 | Automobile mechanics | 728 | 178 |
|  | 7656 | Shoe machine operators and tenders | 454 | 32 |
|  | 6115 | Automotive body and related repairers | 451 | 41 |
|  | 7659 | Miscellaneous textile machine operators and tenders | 424 | 42 |
|  | 6442 | Painters (construction and maintenance) | 378 | 47 |
|  | 5730 | Timber cutting and related occupations | 369 | 97 |
|  | 7661 | Cementing and gluing machine operators and tenders | 360 | 13 |
|  | 7679 | Miscellaneous machine operators and tenders, NEC | 320 | 94 |
|  | 6000 | Supervisors; mechanics and repairers | 301 | 102 |
|  | 7669 | Coating, painting, and spraying machine operators | 274 | 13 |

NEC: not elsewhere classified.

Supplemental Table 6. The correlation between CANJEM-based BTX metrics and Hybrid BTX metrics exposure estimates

|  | Hybrid BTX metrics  (No Lag) | Hybrid BTX metrics  (Lagged 20-Years) |
| --- | --- | --- |
| CANJEM-based BTX metrics (No Lag) | 0.5811 | 0.56 |
|  | <.0001 | <.0001 |
| CANJEM-based BTX metrics (Lagged 20-Years) | 0.56 | 0.5825 |
|  | <.0001 | <.0001 |

| Supplemental Table 7. Odds ratios (ORs) and 95% confidence intervals (CIs) for ever exposure to combined benzene, toluene and xylene (BTX) exposure from job-exposure matrix and with additional expert review and risk of bladder cancer for females only (probability≥50%) | | | | | | | | | | | | | | | | |
| --- | --- | --- | --- | --- | --- | --- | --- | --- | --- | --- | --- | --- | --- | --- | --- | --- |
|  |  | **CANJEM-based BTX metrics** | | | | | | |  | **Hybrid BTX metrics** | | | | | | |
|  |  | No lag | | |  | Lagged 20-Years | | |  | No lag | | |  | Lagged 20-Years | | |
|  |  | Ca | Co | ORs* (95%CI) |  | Ca | Co | ORs* (95%CI) |  | Ca | Co | ORs* (95%CI) |  | Ca | Co | ORs* (95%CI) |
| **Ever exposed** | | |  |  |  |  |  |  |  |  |  |  |  |  |  |  |
|  | None | 29 | 65 | Ref |  | 51 | 95 | Ref |  | 44 | 91 | Ref |  | 65 | 122 | Ref |
|  | Uncertain | 218 | 283 | 1.72 (1.03, 2.88) |  | 199 | 254 | 1.44 (0.93, 2.22) |  | 200 | 255 | 1.50 (0.97, 2.34) |  | 182 | 226 | 1.41 (0.95, 2.09) |
|  | Exposed | 25 | 23 | 2.40 (1.09, 5.25) |  | 22 | 22 | 1.86 (0.87, 3.97) |  | 28 | 25 | 2.39 (1.18, 4.85) |  | 25 | 23 | 2.04 (1.01, 4.15) |

Ca: cases, Co: controls

*Adjusted for age, smoking status, state, race, ethnicity (Hispanic) and non-solvent exposed high-risk occupations for bladder cancer.

Supplemental Table 8. Top 10 jobs with most total cumulative exposure for CANJEM-based BTX metrics, and hybrid BTX metrics at 50% probability among females

| SOC80  Code | SOC title | Total Cumulative  Exposure | Total # Jobs |
| --- | --- | --- | --- |
| **CANJEM-based BTX metrics** | |  |  |
| 7656 | Shoe machine operators and tenders | 424 | 23 |
| 7661 | Cementing and gluing machine operators and tenders | 224 | 6 |
| 6854 | Shoemakers and leather workers and repairers | 135 | 7 |
| 7720 | Assemblers | 122 | 56 |
| 4030 | Supervisors; sales occupations, retail | 106 | 50 |
| 7753 | Hand cutting and trimming occupations | 103 | 8 |
| 7759 | Miscellaneous hand working occupations | 79 | 13 |
| 7659 | Miscellaneous textile machine operators and tenders | 63 | 7 |
| 8769 | Manual occupations, NEC | 54 | 32 |
| 7654 | Textile cutting machine operators and tenders | 53 | 3 |
|  |  |  |  |
| **Hybrid BTX metrics** | |  |  |
| 7656 | Shoe machine operators and tenders | 367 | 23 |
| 7661 | Cementing and gluing machine operators and tenders | 267 | 6 |
| 6854 | Shoemakers and leather workers and repairers | 135 | 7 |
| 7720 | Assemblers | 106 | 56 |
| 7753 | Hand cutting and trimming occupations | 103 | 8 |
| 7759 | Miscellaneous hand working occupations | 79 | 13 |
| 6179 | Mechanics and repairers, not elsewhere classified | 67 | 1 |
| 7654 | Textile cutting machine operators and tenders | 53 | 3 |
| 7678 | Slicing and cutting machine operators and tenders | 32 | 5 |
| 7657 | Pressing machine operators | 32 | 7 |

NEC: not elsewhere classified.
